# Prevalence and determinants of hyperemesis gravidarum among pregnant women in Ethiopia: A systematic review and meta-analysis

**DOI:** 10.1371/journal.pone.0314783

**Published:** 2024-12-03

**Authors:** Girma Alemayehu Beyene, Nitsuh D. Ayele, Abebaw Wasie Kasahun

**Affiliations:** 1 Department of Public Health, College of Medicine and Health Sciences, Wolkite University, Welkite, Ethiopia; 2 Department of Internal Medicine, College of Medicine and Health Sciences, Wolkite University, Welkite, Ethiopia; Osun State University, NIGERIA

## Abstract

**Background:**

Studies focusing on the occurrence and correlates of hyperemesis gravidarum in Ethiopia have reported varied values in different regions of the country. Additionally, there is no systematic review and meta-analysis summarizing the prevalence of hyperemesis gravidarum and its determinants in Ethiopia. Hence, this systematic review and meta-analysis aimed to estimate the overall prevalence of hyperemesis gravidarum and explore its determinants in Ethiopia.

**Methods:**

Cross-sectional or case-control studies conducted in Ethiopia, written in English, and reporting the prevalence or the determinant of hyperemesis gravidarum among pregnant women were included in the review. International databases (PubMed, Scopus, Cochrane Library, Google Scholar, Science Direct, African Journal Online, Directory of Open Access Journal, and African Index Medicus) and Ethiopian university repositories (Jimma, Addis Ababa, Haramaya, Hawassa, and Gondar Universities) were searched from September 4–15, 2023, to identify articles published on the topic. The pooled prevalence of hyperemesis gravidarum with a 95% confidence interval was presented using the forest plots. The heterogeneity of the studies was checked by I^2^ with its corresponding p-values and the Galbraith plot. Subgroup analysis and meta-regression were performed to identify sources of heterogeneity. Funnel plot, Egger, and Begg’s tests were used to assess publication bias.

**Results:**

A total of 11 articles with a 3510-sample size were included in this systematic review and meta-analysis. The pooled prevalence of hyperemesis gravidarum among pregnant women in Ethiopia was 7.12% with a 95% CI (4.09–10.15) and a high level of heterogeneity (I^2^ = 86.5%, p<0.001). Subgroup analyses revealed the overall prevalence of hyperemesis gravidarum was highest in the Amhara region with 11.30%, 95% CI (8.20–14.40), and lowest in Oromia with 3.40%, 95% CI (1.94–4.85). Having a previous history of hyperemesis gravidarum (POR = 3.828, 95% CI: 1.673–5.983), being in the first trimester of pregnancy (POR = 8.476, 95% CI: 5.047–11.905), and *Helicobacter pylori* infection (POR = 3.924, 95% CI: 2.027–5.821) were found to be significantly associated with hyperemesis gravidarum in Ethiopia.

**Conclusion:**

The prevalence of hyperemesis gravidarum among pregnant women in Ethiopia is high. Targeting pregnant women in the first trimester, with a previous history of hyperemesis gravidarum, and those with *Helicobacter pylori* infection during prenatal counseling on how to manage and reduce hyperemesis gravidarum is very helpful to avert related complications.

**Registration:**

The review was registered in the International Prospective Register of Systematic Reviews (PROSPERO) with the registration number “CRD42023461808”, on September 19, 2023.

## Introduction

Nausea and vomiting in pregnancy (NVP) is a common disorder characterized by symptoms of nausea, vomiting, or dry retching commencing in the first trimester without an identifiable cause other than pregnancy [1]. It has a complex and unclear origin that involves genetic, hormonal, endocrine, gastrointestinal, immune, metabolic, environmental, and psychosocial factors [2]. Hyperemesis gravidarum (HG) is the most severe form of unrelenting nausea and vomiting that leads to dehydration, electrolyte and metabolic imbalance, ketonuria, significant weight loss of more than 5% of the pre-pregnancy weight, and impaired daily functioning [3–7].

HG is affecting 0.3%-3.6% of pregnant women globally [8]. The prevalence is notably high in China (8.9%), and Northeast Nigeria (44.9%), and varies in Ethiopia from 3.2% in Hararghe to 11.7% in Addis Ababa [5, 9–15].

Hyperemesis gravidarum is more likely in women who are young, single, primigravida, carrying a girl or a molar pregnancy, have a previous or family history of HG, have a history of abortion or multiple gestations [5, 7, 9–11, 14–28]. Additionally, gestational age, maternal nutritional status, history of gastrointestinal disease, asthma, previous history of urinary infection, *H*. *pylori* infection, and stress were factors reported by literature to be associated with hyperemesis gravidarum [10, 12, 24, 26, 29–37].

Women with hyperemesis are at increased risk of having medical and obstetric problems during pregnancy as well as a higher rate of maternal and fetal complications. These include failure to gain weight, hypertensive disorders, anemia, preterm birth, low birth weight, post-partum hemorrhage, placental abruption, and neonatal intensive care unit admission [38–44]. It may also increase the likelihood of psychological and emotional distress during and after pregnancy, and some women may have suicidal thoughts or consider ending their wanted pregnancies [45–52]. The health-related quality of life of women with moderate to severe NVP is comparable to those with breast cancer or myocardial infarction [53, 54]. Children exposed to HG in utero may have increased risks of various health problems later in life, such as hormonal imbalances, metabolic disorders, growth and neurodevelopmental impairment [41, 55–61].

A comprehensive understanding of the condition, improving healthcare provider awareness, and providing appropriate psychological support, prompt and timely recognition, identification, and treatment are essential to minimize or prevent associated long-term maternal and fetal morbidity/complications [7, 42, 62, 63].

Studies focusing on the occurrence and correlates of hyperemesis gravidarum in Ethiopia have reported varied values in different regions of the country. Additionally, there is no systematic review and meta-analysis summarizing the prevalence of hyperemesis gravidarum and its determinants in Ethiopia. Hence, the objective of this systematic review and meta-analysis is to estimate the overall prevalence of hyperemesis gravidarum and explore its determinants in Ethiopia. Understanding the burden of the problem and identification of the determinant factors are important in pre-pregnancy counseling to detect early and minimize related complications and adverse outcomes. The findings of this systematic review and meta-analysis will be used by the concerned stakeholders to reduce the prevalence of hyperemesis gravidarum and design evidence-based interventions.

## Methods

### Reporting and protocol registration

The research database and Prospective Register of Systematic Reviews (PROSPERO) have been searched to check if the topic has been reviewed previously or if an ongoing review exists to avoid re-inventing the wheel. Hence, no systematic review or meta-analysis on this topic was published or registered. The updated Preferred Reporting Items for Systematic Reviews and Meta-analysis guideline (PRISMA-P) protocol was followed for conducting and reporting this systematic review and meta-analysis (S1 Table). The review protocol has been registered in PROSPERO, the University of York Centre for Reviews and Dissemination, with the registration number “CRD42023461808”, as of September 19, 2023.

### Eligibility criteria

Cross-sectional or case-control studies conducted in Ethiopia, written in English, and reporting the prevalence or determinant factors of hyperemesis gravidarum among pregnant women were included in the review. Cross-sectional studies were used to calculate the pooled prevalence of hyperemesis gravidarum. Duplicated studies, articles without abstract and full text, and studies that do not report the odds ratio of the factors were excluded.

### Information source

International online databases (such as PubMed, Scopus, Cochrane Library, Google Scholar, Science Direct, African Journal Online, Directory of Open Access Journal, and African Index Medicus) and Ethiopian university repositories (Jimma, Addis Ababa, Haramaya, Hawassa, and Gondar Universities) were searched for articles on prevalence and determinants of hyperemesis gravidarum from September 4–15, 2023.

### Search strategy

**Fig 1:**
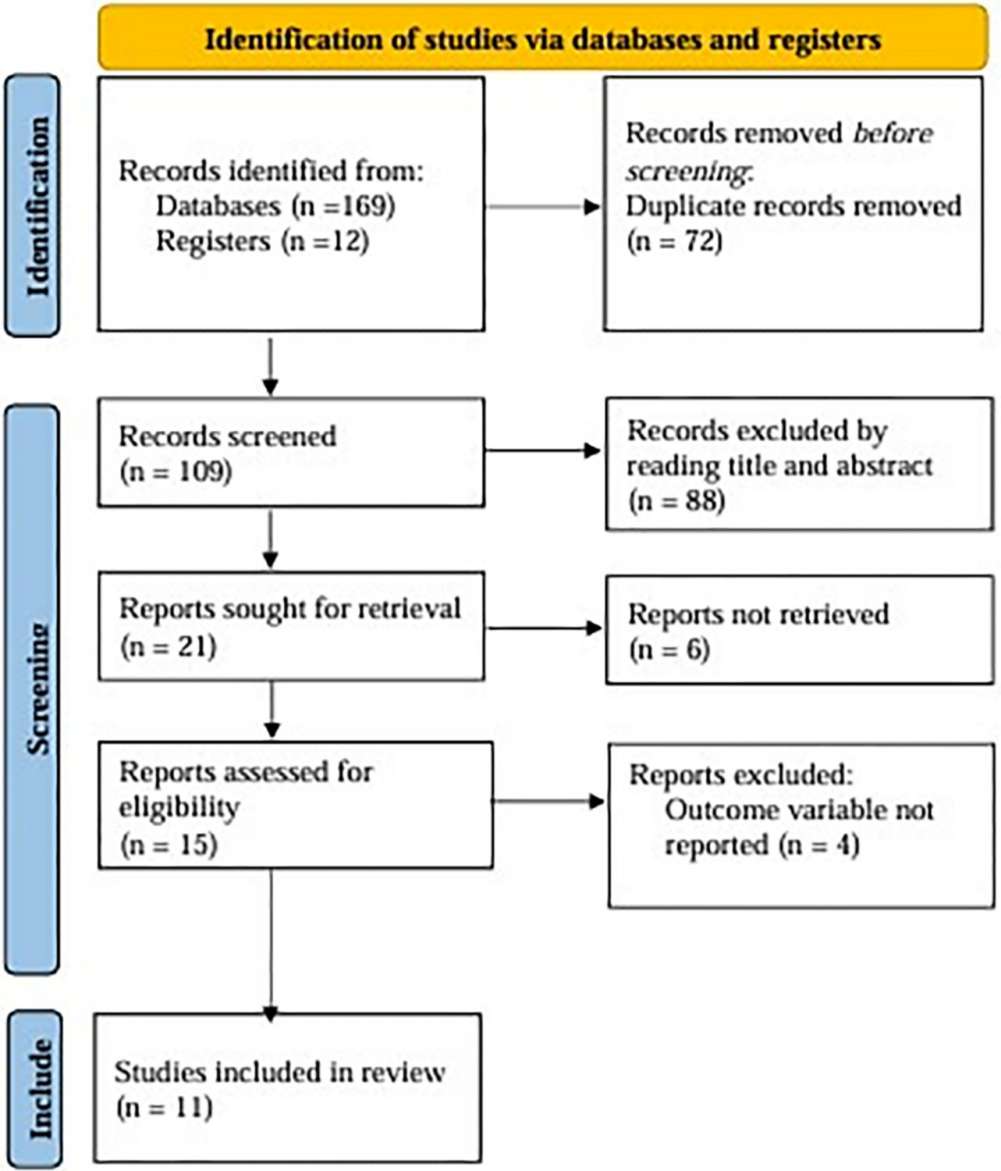
Flow chart showing the selection of studies for the systematic review and meta-analysis of prevalence and determinants of hyperemesis gravidarum among pregnant women in Ethiopia.

Search terms based on Population, Intervention, Comparison, Outcome (PICO) questions, and medical subject heading (MeSH) terms; Boolean operators "AND" and "OR" were used. “Hyperemesis gravidarum” OR “hyperemesis” AND “gravidarum” OR “nausea and vomiting in pregnancy” OR “nausea” OR “vomiting” OR “pregnancy complications” OR “maternal morbidity” AND “prevalence” OR “magnitude” OR “burden” AND “determinants” OR “risk factors” OR “associated factors” OR “predictors” AND “Ethiopia” were used to search articles on the databases. In addition to consultation with the subject matter experts, the list of references for the included articles was also searched to find potential articles (S2 Table). EndNote™ 20 reference manager software was used to organize search results and remove duplicate articles. The study selection process was presented in a PRISMA flowchart (Fig 1).

### Selection process

To determine the eligibility of the studies for the review, independent reviewers applied the pre-defined inclusion criteria. Two authors (GA & AW) independently screened and selected the articles, and resolved any disagreement by consulting with a third author (ND).

### Data collection process

Once eligible studies were identified from the databases, the data extraction format was prepared on a Microsoft Excel spreadsheet and used to extract the data from the articles. The data extraction format included the name of the author, the year it was published, the region and specific location where the study was conducted, the type of study design employed, and the sample size of participants. The prevalence of hyperemesis gravidarum and the odds ratio of the selected variables were also recorded.

### Outcome measurement

This study considered two major outcomes of interest. First, the percentage of pregnant women who suffered from hyperemesis gravidarum. The second one was the factors that influenced the occurrence of hyperemesis gravidarum, expressed by the odds ratio with the confidence intervals. Although there is no clear consensus on how to define hyperemesis gravidarum, all the studies that were included in this review used the same criteria: “Pregnant women who had frequent nausea and vomiting not related to other causes and/or who had dehydration, electrolyte abnormalities, ketonuria on urine analysis, or weight loss of at least 5% of their pre-pregnancy weight.”

### Study risk of bias assessment

To assess the risk of bias in the included studies, Joanna Briggs Institute’s (JBI) quality appraisal tool was used, with eight criteria rated as yes, no, not applicable, and classified risks of bias as low, moderate, or high.

### Effect measures

The effect measures for the synthesis and presentation of the results were the precomputed prevalence and odds ratio, along with their respective confidence intervals.

### Synthesis methods

We calculated the pooled prevalence of hyperemesis gravidarum by analyzing cross-sectional studies that measured its occurrence. The odds ratio of the factors associated with hyperemesis gravidarum was determined from both cross-sectional and case-control studies. The data from the studies were extracted using a Microsoft Excel 2021 spreadsheet, and then meta-analyses were conducted using Stata MP 17. To address the missing values identified in the primary data for key outcomes or variables essential to the meta-analysis, we contacted the authors of the original studies. We minimized the risk of introducing bias by obtaining the missing information directly from the source.

The estimated prevalence of each study and analysis of determinant factors were presented using the forest plots with a 95% confidence interval. The heterogeneity of the studies was checked by I^2^ with its corresponding p-values and the Galbraith plot. Analysis of the random effect model was used because of the high evidence of heterogeneity. To explore possible sources of heterogeneity among the studies, subgroup analyses and meta-regression were performed. Sensitivity analysis was conducted to understand the effect of a single study on the pooled estimate value. The results of the study were presented using narration, tables, and graphs.

### Publication/reporting bias assessment

Funnel plot and Egger and Begg’s regression asymmetry tests with a p-value of <0.05 were used to assess and decide reporting or publication bias.

## Results

### Study selection

The PRISMA flowchart (Fig 1) shows the study selection process.

### Study characteristics

A total of 11 articles with a 3510-sample size were included in this systematic review and meta-analysis. Six of the included studies were cross-sectional, and the remaining five were case-control studies. The pooled prevalence was calculated from the cross-sectional studies (S3 Table), and the factors were identified from both cross-sectional and case-control studies (S4 Table). Concerning their regional distribution, four were conducted in the Oromia region, three in Addis Ababa city, two in the Amhara region, one in South Ethiopia, and one in the Tigray region (Table 1).

**Table 1:** Study characteristics included in the systematic review and meta-analysis.

S. No	Author	Year	Region	Study area	Design	Sample size	Quality assessment
1	Segni et.al	2016	Oromia	Jimma	Cross-sectional	102	Low risk
2	Gelmesa et.al	2021	Oromia	Hararghe	Cross-sectional	495	Low risk
3	Kuma et.al	2013	Addis Ababa	TAH, GMH, SPHMMC**†**	Cross-sectional	384	Low risk
4	Fessehaye et.al	2021	Addis Ababa	SPHMMC	Cross-sectional	350	Low risk
5	Kejela et.al	2018	South	Arbaminch	Cross-sectional	183	Low risk
6	Adane et.al	2023	Amhara	South Wollo	Cross-sectional	355	Low risk
7	Teferi et.al	2021	Addis Ababa	Kirkos sub-city	Case-control	150	Low risk
8	Mekonnen et.al	2018	Oromia	Bale	Case-control	423	Low risk
9	Solomon et.al	2023	Oromia	Guji, West Guji & Borana	Case-control	309	Low risk
10	Asrade et.al	2023	Amhara	Bahirdar	Case-control	444	Low risk
11	Tefera et.al	2021	Tigray	Mekelle	Case-control	315	Low risk

**†**TAH: Tikur Anbessa Hospital, GMH: Gandi Memorial Hospital, SPHMMC: St. Paul Hospital Millennium Medical College

### Risk of bias in studies

The risk of bias in the included studies was evaluated by Joanna Briggs Institute’s (JBI) quality appraisal tools, using eight criteria rated as yes, no, not applicable, and classified risks of bias as low, moderate, or high. Thus, all the included articles were assessed and found to have a low risk of bias. The details of the risk of bias assessment for the included studies are displayed below (Table 2).

**Table 2:** Joanna Briggs Institute’s (JBI) quality appraisal result of the included studies.

Studies	Clear inclusion & exclusion criteria	Description of the study subject and setting	Valid, reliable method to measure exposure	Standard criteria for measurement of condition	Identification of confounding factors	Strategies to deal with confounding factors	Valid, reliable method to measure outcomes	Appropriate statistical analysis	Total score	Risk of bias
Segni et.al	Yes	Yes	Yes	Yes	Yes	Yes	Yes	No	7	Low
Gelmesa et.al	Yes	Yes	Yes	Yes	Yes	Yes	Yes	Yes	8	Low
Kuma et.al	Yes	Yes	Yes	Yes	Yes	Yes	Yes	No	7	Low
Fessehaye et.al	Yes	Yes	Yes	Yes	Yes	Yes	Yes	Yes	8	Low
Kejela et.al	Yes	Yes	Yes	Yes	NA	NA	Yes	Yes	6	Low
Adane et.al	Yes	Yes	Yes	Yes	Yes	Yes	Yes	Yes	8	Low
Teferi et.al	Yes	Yes	Yes	Yes	Yes	Yes	Yes	No	7	Low
Mekonnen et.al	No	Yes	Yes	Yes	Yes	Yes	Yes	No	6	Low
Solomon et.al	Yes	Yes	Yes	Yes	Yes	Yes	Yes	Yes	8	Low
Asrade et.al	Yes	Yes	Yes	Yes	Yes	Yes	Yes	Yes	8	Low
Tefera et.al	Yes	Yes	Yes	Yes	Yes	Yes	Yes	Yes	8	Low

### Prevalence of hyperemesis gravidarum in Ethiopia

**Fig 2:**
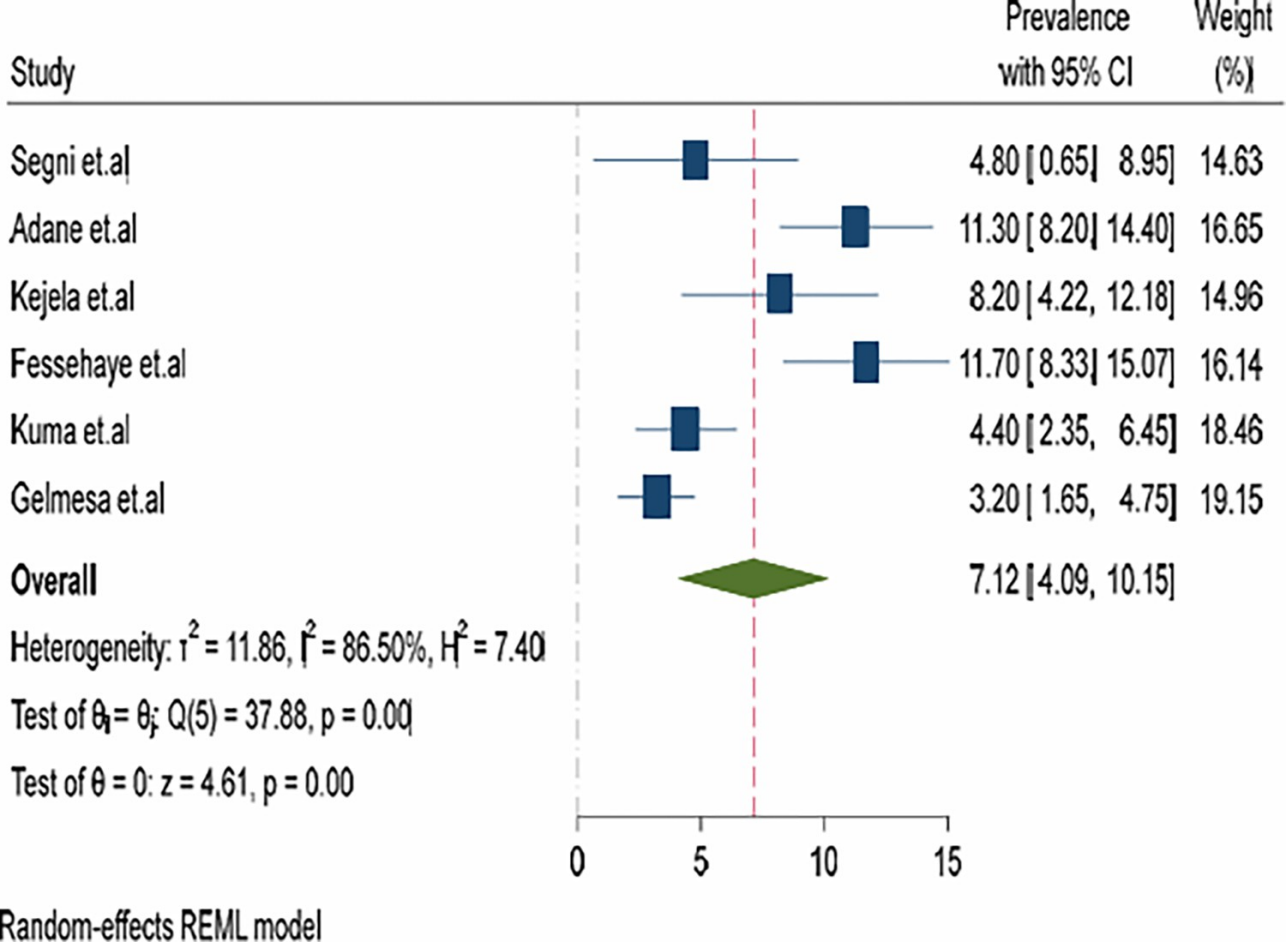
Pooled prevalence of hyperemesis gravidarum among pregnant women in Ethiopia, 2023.

Based on the random effect model, the pooled prevalence of hyperemesis gravidarum among pregnant women in Ethiopia is 7.12% with a 95% CI (4.09–10.15) and a high level of heterogeneity, (I^2^ = 86.5%, p<0.001). The graphical display of the prevalence of the individual studies and the result of the synthesis is presented in the forest plot (Fig 2).

### Heterogeneity analysis

**Fig 3:**
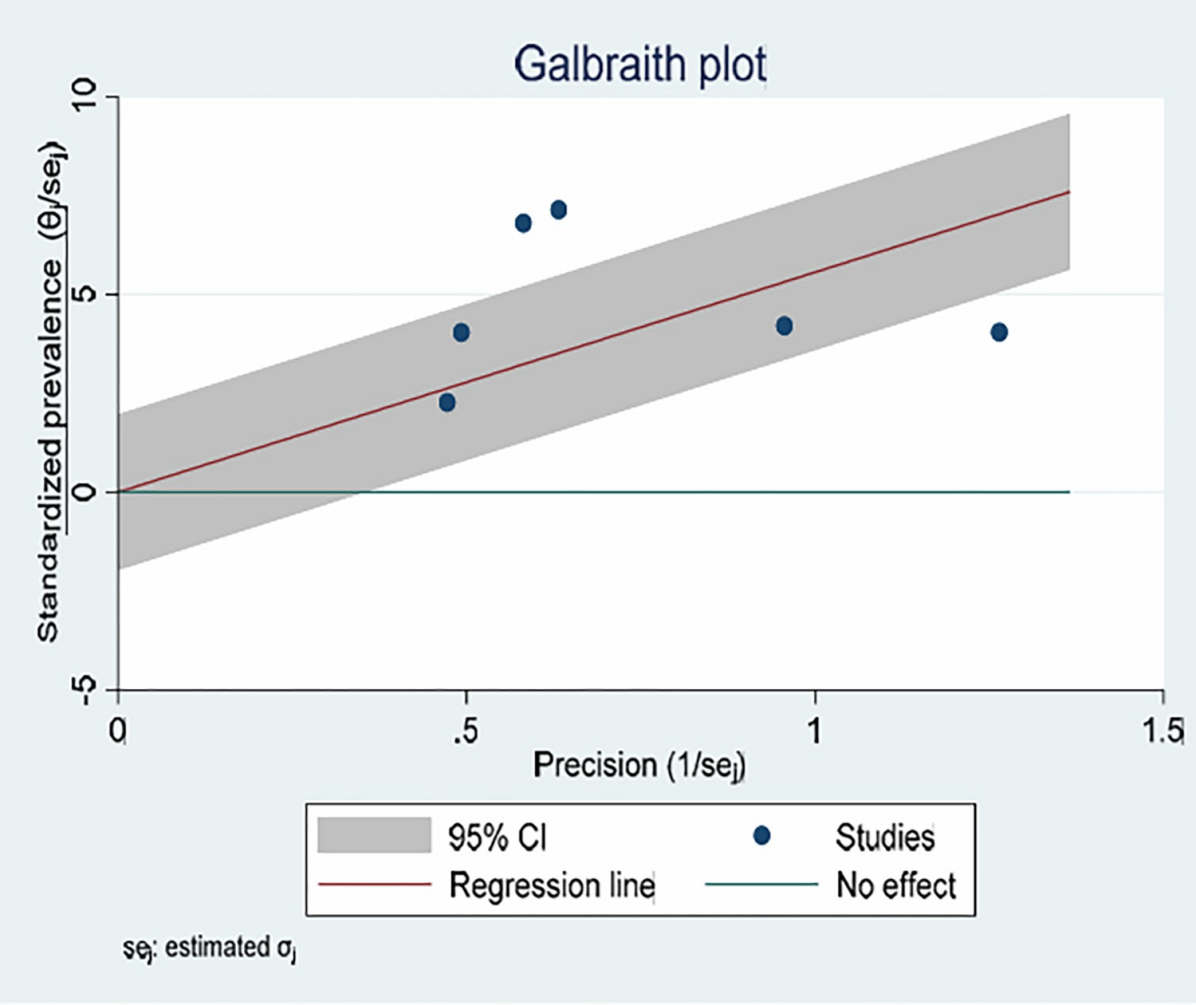
Galbraith plot displaying heterogeneity of studies reporting the prevalence of hyperemesis gravidarum among pregnant women in Ethiopia, 2023.

As observed from I^2^ of 86.5% as displayed on the forest plot and the Galbraith plot (Fig 3) there is high heterogeneity among the included studies.

**Fig 4:**
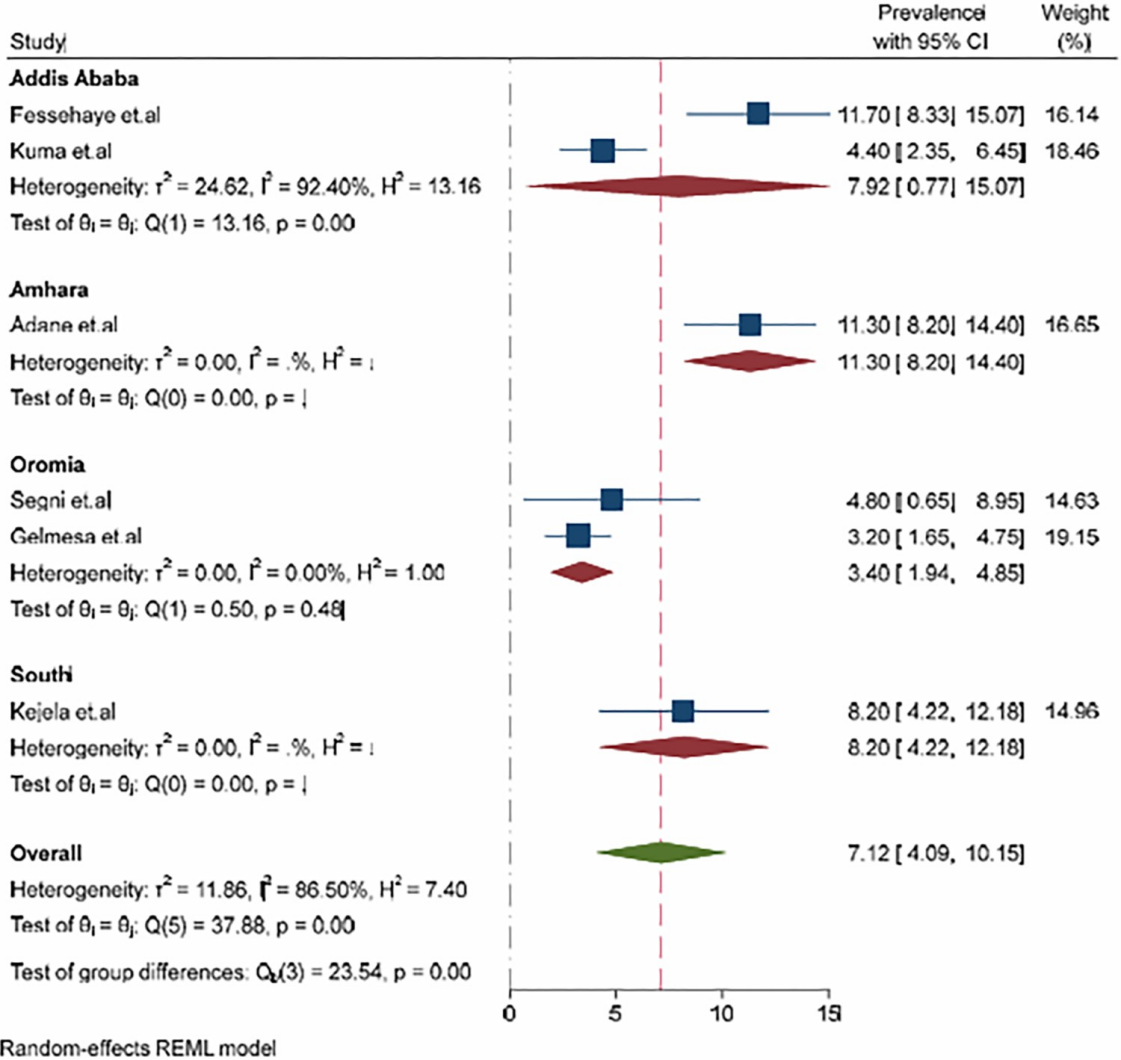
Forest plot presenting sub-group analysis of hyperemesis gravidarum among pregnant women by region in Ethiopia, 2023.

To identify the source of heterogeneity, subgroup analyses of the prevalence of hyperemesis gravidarum were further calculated based on the geographical locations of the included studies. Accordingly, the overall prevalence of hyperemesis gravidarum is highest in the Amhara region with 11.30%, 95% CI (8.20–14.40), and lowest in the region of Oromia with 3.40%. 95% CI (1.94–4.85) (Fig 4).

We also performed a meta-regression analysis to examine the effects of the year of publication and the sample size. However, none of these variables showed statistically significant association with a p-value of 0.150 for the publication year and 0.432 for the sample size (Table 3).

**Table 3:** Meta-regression analysis of hyperemesis gravidarum among pregnant women in Ethiopia, 2023.

_meta_es	Coefficient	Std. error	z	P>|z|	[95% conf. interval]	
Year	0.666	0.46	1.44	0.150	-0.24	1.57
Sample size	-0.009	0.01	-0.79	0.432	-0.03	0.01
_cons	-1333.96	933.25	-1.43	0.153	-3163.09	495.18

### Determinants of hyperemesis gravidarum in Ethiopia

The pooled analysis of the studies revealed that having a previous history of hyperemesis gravidarum, being in the first trimester of pregnancy, and having a *Helicobacter pylori* infection were determinants associated with hyperemesis gravidarum. Pregnant women who have a previous history of hyperemesis gravidarum are almost four times more likely to have hyperemesis gravidarum compared to those who do not have a previous history of hyperemesis gravidarum, with a pooled OR of 3.83 and a 95% CI of 1.67–5.98. Compared to those who are in the third trimester of pregnancy, pregnant women in the first trimester are more than eight times more likely to have hyperemesis gravidarum, with a pooled OR of 8.48, 95% CI (5.05–11.91). Pregnant women who have a *Helicobacter pylori* infection are almost four times more likely to have hyperemesis gravidarum compared to those who are negative for *Helicobacter pylori*, with a pooled OR of 3.92 and a 95% CI of 2.03–5.82 (Table 4).

**Table 4:** Determinants of hyperemesis gravidarum among pregnant women in Ethiopia, 2023.

Variables	Author	AOR	95% CI	Pooled OR	95% CI of Pooled OR
Previous History of HG	Mekonnen et.al	3.49	1.90–6.43	3.83	1.67–5.98
Previous History of HG	Adane et.al	10.9	2.46–48.44		
Previous History of HG	Solomon et.al	6.66	2.57–17.27		
First trimester	Mekonnen et.al	8.90	7.00–14.76	8.48	5.05–11.91
First trimester	Tefera et.al	6.01	1.87–19.26		
First trimester	Asrade et.al	9.30	2.88–30.07		
*Helicobacter Pylori* infection	Tefera et.al	3.50	1.92–6.39	3.92	2.03–5.82
*Helicobacter Pylori* infection	Teferi et.al	12.87	5.32–31.32		
*Helicobacter Pylori* infection	Asrade et.al	4.37	2.01–9.48		

### Publication bias

**Fig 5:**
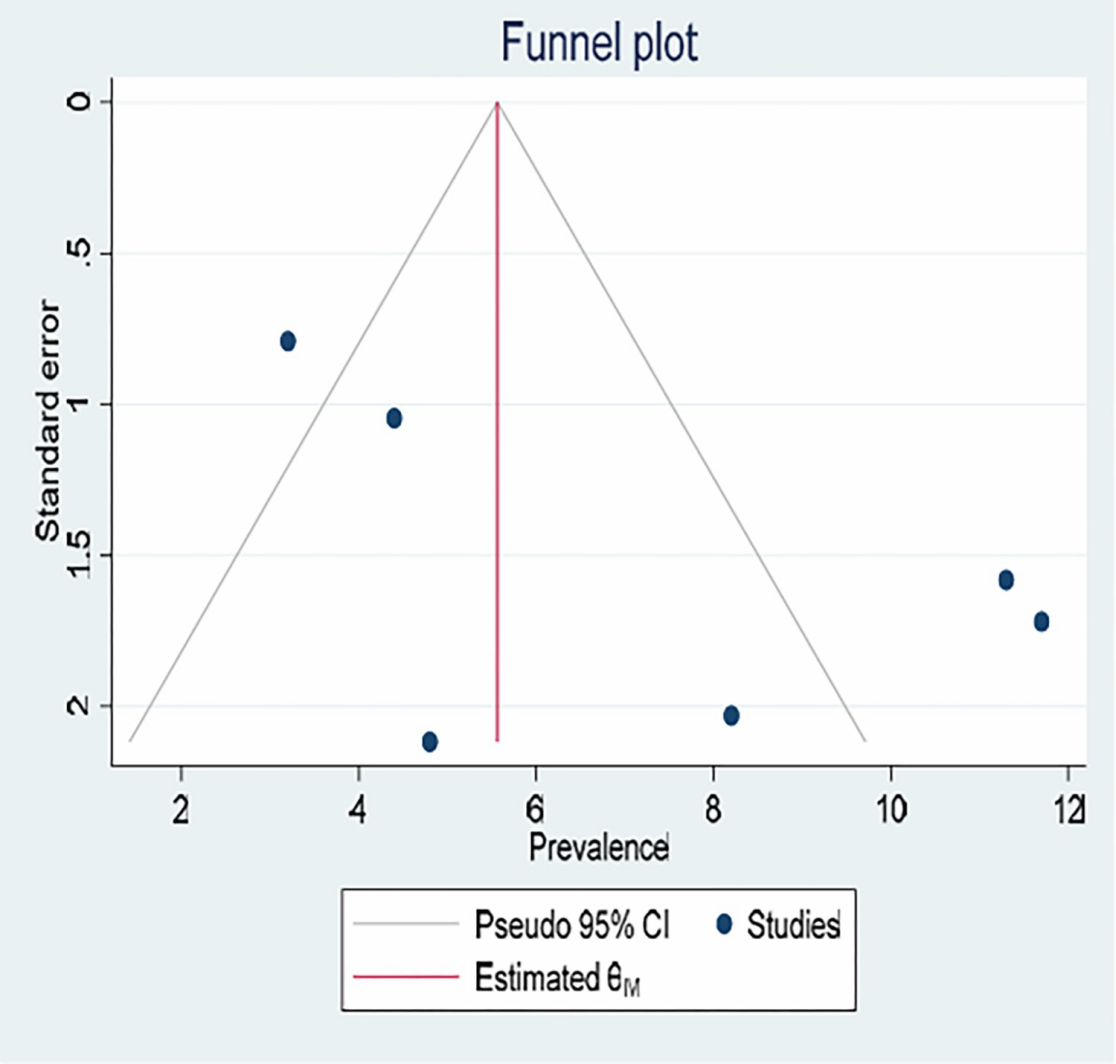
Funnel plot displaying publication bias of studies reporting the pooled prevalence of hyperemesis gravidarum among pregnant women in Ethiopia, 2023.

The presence of reporting or publication bias was evaluated visually using funnel plot asymmetry and statistically using Egger and Begg’s test for small study effects. As observed from the plot, there is some sort of asymmetry, which might be due to the detected heterogeneity or simply by chance. Regression-based Egger’s and Begg’s tests were used to test whether the funnel-plot asymmetry was greater than what had been expected by chance. Thus, Egger’s test (p = 0.2117) and Begg’s test (p = 7071) were not statistically significant, confirming no reporting bias and indicating the funnel plot asymmetry might be observed by chance (Fig 5).

### Sensitivity analysis

**Fig 6:**
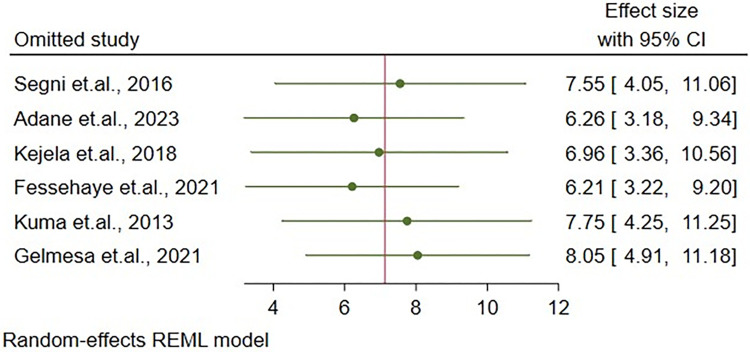
Sensitivity analysis using leave-one-out meta-analysis of the prevalence of hyperemesis gravidarum among pregnant women in Ethiopia, 2023.

According to the result of sensitivity analysis, using leave-one-out meta-analysis, no single study in this systematic review and meta-analysis dominated the overall prevalence of hyperemesis gravidarum in Ethiopia (Fig 6).

## Discussion

This systematic review and meta-analysis estimated the overall prevalence of hyperemesis gravidarum in Ethiopia and explored its determinants. Hence, 7.12%, with a 95% CI (4.09–10.15), of pregnant women have hyperemesis gravidarum in Ethiopia. Subgroup analyses, based on the geographical locations where the included studies were conducted, revealed the highest and lowest prevalence of Hyperemesis gravidarum documented in the Amhara and Oromia regions, respectively. This is much lower than the study conducted in Nigeria, where almost half of the study participants had hyperemesis gravidarum, and more than the study conducted in Finland, where 1.3% of pregnancies resulting in delivery had been diagnosed with hyperemesis gravidarum [64]. The difference might be due to how hyperemesis was measured. The study in Finland measured hyperemesis gravidarum as an incidence among pregnancies that ended in delivery. However, the studies included in this review measured hyperemesis among all pregnant women, irrespective of their outcomes [14, 65].

The prevalence of hyperemesis gravidarum (HG) observed in our meta-analysis is lower than the study conducted at comprehensive specialized hospitals in northwest Ethiopia which reported 16.8% prevalence. This discrepancy may be attributed to the study setting, as the latter was conducted at specialized hospitals where most patients were referred, potentially leading to a higher concentration of severe cases [66].

Having a previous history of hyperemesis gravidarum, being in the first trimester of pregnancy, and *helicobacter pylori* infection were determinant factors associated with hyperemesis gravidarum among pregnant women in Ethiopia. Pregnant women who have a previous history of hyperemesis gravidarum are more likely to experience hyperemesis gravidarum compared to those who do not have a previous history of hyperemesis gravidarum. This finding was in line with the studies conducted in Australia, Finland, Nigeria, and Ethiopia, where women with a previous history of hyperemesis gravidarum are more likely to experience hyperemesis gravidarum. The increased risk of hyperemesis gravidarum in subsequent pregnancies among women with a prior history of the condition may be attributed to several factors. These may include psychological stress due to the anticipation of recurrent HG, the persistence of risk factors from previous pregnancies, and the possibility of genetic predisposition to HG [14, 17, 21, 66, 67].

Compared to those who are in the third trimester of pregnancy, pregnant women in the first trimester are more likely to have hyperemesis gravidarum. The same finding was observed in a study conducted in Indonesia, where women in their first trimester were more likely to be affected by hyperemesis gravidarum [68]. This might be because the level of human chorionic gonadotropin is higher in the first trimester of pregnancy, resulting in excess nausea and vomiting. This justification is supported by other related literature [69–71].

Pregnant women who have a *Helicobacter pylori* infection are more likely to have hyperemesis gravidarum compared to those who are negative for *Helicobacter pylori*. Similar results were reported by studies conducted in Turkiye, Iraq, Pakistan, Egypt, and Ethiopia, where *Helicobacter pylori* infection was found to have a statistically significant association with hyperemesis gravidarum. The potential mechanisms underlying this association may include the inflammatory response triggered by *H*. *pylori* infection in the gastric mucosa, leading to gastrointestinal disturbances such as gastritis and peptic ulcers. This inflammation can exacerbate symptoms of nausea and vomiting. Additionally, *H*. *pylori* infection may be linked to elevated levels of gastrin hormone, resulting in increased gastric acidity. The infection may also induce the release of cytokines such as interleukin-6 and tumor necrosis factor-alpha, which contribute to gastrointestinal symptoms. These cytokines can affect the central nervous system and gastrointestinal motility [72–75]. Hence, pregnant women with persistent hyperemesis gravidarum should be investigated for active *Helicobacter pylori* infection, and eradication therapy has to be initiated [29, 31–33, 35, 36].

While our study provides valuable insights, it is not without limitations. Firstly, the small sample size in some included studies may affect the meta-analysis findings. Secondly, the inclusion of factors identified from cross-sectional studies introduces the potential for confounding variables to influence the estimates. The possibility of unexamined confounders may contribute to the observed heterogeneity.

## Conclusion

A high proportion of pregnant women in Ethiopia experience hyperemesis gravidarum. The pooled prevalence of hyperemesis gravidarum among pregnant women in Ethiopia is high. Having a previous history of hyperemesis gravidarum, being in the first trimester of pregnancy, and *helicobacter pylori* infection were significant determinant factors associated with hyperemesis gravidarum among pregnant women in Ethiopia.

Hence, targeting pregnant women in the first trimester with a previous history of hyperemesis gravidarum and those with *Helicobacter pylori* infection during prenatal counseling on how to manage and reduce hyperemesis gravidarum is very helpful to avert related complications.

## Supporting information

S1 Table PRISMA checklist for reporting hyperemesis gravidarum among pregnant women in Ethiopia, 2023. (DOCX)

S2 Table Total studies identified and retrieved in the literature search, and assessed for eligibility for the systematic review and meta-analysis of hyperemesis gravidarum among pregnant women in Ethiopia, 2023. (DOCX)

S3 Table Studies included in the analysis of prevalence hyperemesis gravidarum among pregnant women in Ethiopia, 2023. (DOCX)

S4 Table Studies included in the analysis of determinants of hyperemesis gravidarum among pregnant women in Ethiopia, 2023. (DOCX)

## Abbreviations

HG: Hyperemesis gravidarum
NVP: Nausea and vomiting in pregnancy
POR: Pooled Odds Ratio
