# Supplementary material for: Prevalence and determinants of hyperemesis gravidarum among pregnant women in Ethiopia: A systematic review and meta-analysis
Source: PLoS One. 2024 Dec 3;19(12):e0314783. doi: 10.1371/journal.pone.0314783 (PMC11614256; doi:10.1371/journal.pone.0314783)
Supplement: S2 Table — (DOCX) [file pone.0314783.s002.docx]

**S2 Table. Total studies identified and retrieved in the literature search, and assessed for eligibility for the systematic review and meta-analysis of hyperemesis gravidarum among pregnant women in Ethiopia, 2023**

| **S. N** | **Author** | **Year** | **Region** | **Study area** | **Design** | **Remark** |
| --- | --- | --- | --- | --- | --- | --- |
| 1 | Mekonnen et.al | 2018 | Oromia | Bale | Case-control | Included in the analysis |
| 2 | Tefera et.al | 2021 | Tigray | Mekelle | Case-control | Included in the analysis |
| 3 | Adane et.al | 2023 | Amhara | South Wollo | Cross-sectional | Included in the analysis |
| 4 | Asrade et.al | 2023 | Amhara | Bahirdar | Case-control | Included in the analysis |
| 5 | Solomon et.al | 2023 | Oromia | Guji, West Guji & Borana | Case-control | Included in the analysis |
| 6 | Fessehaye et.al | 2021 | Addis Ababa | St. PHMMC | Cross-sectional | Included in the analysis |
| 7 | Teferi et.al | 2021 | Addis Ababa | Kirkos Sub-city | Case-control | Included in the analysis |
| 8 | Segni et.al | 2016 | Oromia | Jimma | Cross-sectional | Included in the analysis |
| 9 | Kejela et.al | 2018 | South | Arbaminch | Cross-sectional | Included in the analysis |
| 10 | Kuma et.al | 2013 | Addis Ababa | TAH, GMH, SPHMMC**†** | Cross-sectional | Included in the analysis |
| 11 | Gelmesa et.al | 2021 | Oromia | Hararghe | Cross-sectional | Included in the analysis |
| 12 | Assefa et.al | 2017 | Addis Ababa | Kirkos Sub-city | Case-control | Excluded because no outcome variable |
| 13 | Bantie et.al | 2018 | Addis Ababa | Gandi Memorial Hospital | Cross-sectional | Excluded because different outcome variable measurement |
| 14 | Ayenew et.al | 2019 | Amhara | Debre Berhan | Cross-sectional | Excluded because no outcome variable |
| 15 | Chekol et.al | 2021 | Gondar | South Gondar | Cross-sectional | Excluded because different outcome variable measurement |

**†**TAH: Tikur Anbessa Hospital, GMH: Gandi Memorial Hospital, SPHMMC: St. Paul Hospital Millennium Medical College
