# Supplementary material for: Prevalence and determinants of hyperemesis gravidarum among pregnant women in Ethiopia: A systematic review and meta-analysis
Source: PLoS One. 2024 Dec 3;19(12):e0314783. doi: 10.1371/journal.pone.0314783 (PMC11614256; doi:10.1371/journal.pone.0314783)
Supplement: S3 Table — (DOCX) [file pone.0314783.s003.docx]

**S3 Table. Studies included in the analysis of prevalence hyperemesis gravidarum among pregnant women in Ethiopia, 2023**

| **S.N** | **Author** | **Year** | **Design** | **Sample size** | **Prevalence** | **LCL** | **UCL** | **Data extractor** | **Date of data extraction** | **Eligibility** |
| --- | --- | --- | --- | --- | --- | --- | --- | --- | --- | --- |
| 1 | Segni et.al | 2016 | Cross-sectional | 102 | 4.8 | 0.65 | 8.95 | GA & AW | 13/09/2023 | Eligible |
| 2 | Adane et.al | 2023 | Cross-sectional | 355 | 11.3 | 8.2 | 14.4 | GA & AW | 13/09/2023 | Eligible |
| 3 | Kejela et.al | 2018 | Cross-sectional | 183 | 8.2 | 4.22 | 12.18 | GA & AW | 13/09/2023 | Eligible |
| 4 | Fessehaye et.al | 2021 | Cross-sectional | 350 | 11.7 | 8.33 | 15.07 | GA & AW | 13/09/2023 | Eligible |
| 5 | Kuma et.al | 2013 | Cross-sectional | 384 | 4.4 | 2.35 | 6.45 | GA & AW | 13/09/2023 | Eligible |
| 6 | Gelmesa et.al | 2021 | Cross-sectional | 495 | 3.2 | 1.65 | 4.75 | GA & AW | 13/09/2023 | Eligible |
