# Supplementary material for: Prevalence and determinants of hyperemesis gravidarum among pregnant women in Ethiopia: A systematic review and meta-analysis
Source: PLoS One. 2024 Dec 3;19(12):e0314783. doi: 10.1371/journal.pone.0314783 (PMC11614256; doi:10.1371/journal.pone.0314783)
Supplement: S4 Table — (DOCX) [file pone.0314783.s004.docx]

**S4 Table. Studies included in the analysis of determinants of hyperemesis gravidarum among pregnant women in Ethiopia, 2023**

| **S.N** | **Factors** | **Year** | **Design** | **Sample size** | **Author** | **AOR** | **LCL** | **UCL** | **Data extractor** | **Date data extracted** | **Eligibility** |
| --- | --- | --- | --- | --- | --- | --- | --- | --- | --- | --- | --- |
| 1 | Urban area | 2018 | Case-control | 423 | Mekonnen et.al | 2.96 | 1.50 | 5.86 | GA & AW | 14/09/2023 | Eligible |
|  | Urban area | 2021 | Case-control | 327 | Tefera et.al | 0.49 | 0.18 | 1.38 | GA & AW | 14/09/2023 | Eligible |
|  | Urban area | 2022 | Case-control | 360 | Ashebir et.al | 2.10 | 1.01 | 4.34 | GA & AW | 14/09/2023 | Eligible |
|  | Urban area | 2023 | Case-control | 444 | Asrade et.al | 2.72 | 1.69 | 4.50 | GA & AW | 14/09/2023 | Eligible |
| 2 | Family history of HG | 2018 | Case-control | 423 | Mekonnen et.al | 1.14 | 0.59 | 2.22 | GA & AW | 14/09/2023 | Eligible |
|  | Family history of HG | 2021 | Case-control | 327 | Tefera et.al | 3.85 | 1.69 | 8.75 | GA & AW | 14/09/2023 | Eligible |
|  | Family history of HG | 2023 | Case-control | 444 | Asrade et.al | 5.02 | 2.59 | 9.69 | GA & AW | 14/09/2023 | Eligible |
|  | Family history of HG | 2023 | Case-control | 309 | Solomon et.al | 2.07 | 1.07 | 4.02 | GA & AW | 14/09/2023 | Eligible |
| 3 | Previous History of HG | 2018 | Case-control | 423 | Mekonnen et.al | 3.49 | 1.90 | 6.43 | GA & AW | 14/09/2023 | Eligible |
|  | Previous History of HG | 2023 | Cross-sectional | 355 | Adane et.al | 10.9 | 2.46 | 48.44 | GA & AW | 14/09/2023 | Eligible |
|  | Previous History of HG | 2023 | Case-control | 309 | Solomon et.al | 6.66 | 2.57 | 17.27 | GA & AW | 14/09/2023 | Eligible |
| 4 | First trimester | 2018 | Case-control | 423 | Mekonnen et.al | 8.90 | 7.00 | 14.76 | GA & AW | 14/09/2023 | Eligible |
|  | First trimester | 2021 | Case-control | 327 | Tefera et.al | 6.01 | 1.87 | 19.26 | GA & AW | 14/09/2023 | Eligible |
|  | First trimester | 2023 | Case-control | 444 | Asrade et.al | 9.30 | 2.88 | 30.07 | GA & AW | 14/09/2023 | Eligible |
| 5 | Second trimester | 2018 | Case-control | 423 | Mekonnen et.al | 9.08 | 2.95 | 27.91 | GA & AW | 14/09/2023 | Eligible |
|  | Second trimester | 2021 | Case-control | 327 | Tefera et.al | 4.73 | 1.59 | 14.00 | ND & AW | 15/09/2023 | Eligible |
|  | Second trimester | 2023 | Case-control | 444 | Asrade et.al | 4.79 | 1.45 | 15.80 | ND & AW | 15/09/2023 | Eligible |
| 6 | Primigravidity | 2021 | Case-control | 327 | Tefera et.al | 1.31 | 0.32 | 5.38 | ND & AW | 15/09/2023 | Eligible |
|  | Primigravidity | 2022 | Case-control | 360 | Ashebir et.al | 1.55 | 0.86 | 2.79 | ND & AW | 15/09/2023 | Eligible |
|  | Primigravidity | 2023 | Case-control | 444 | Asrade et.al | 6.18 | 3.13 | 12.20 | ND & AW | 15/09/2023 | Eligible |
| 7 | H. Pylori infection | 2021 | Case-control | 327 | Tefera et.al | 3.50 | 1.92 | 6.39 | ND & AW | 15/09/2023 | Eligible |
|  | H. Pylori infection | 2021 | Case-control | 150 | Teferi et.al | 12.87 | 5.32 | 31.32 | ND & AW | 15/09/2023 | Eligible |
|  | H. Pylori infection | 2023 | Case-control | 444 | Asrade et.al | 4.37 | 2.01 | 9.48 | ND & AW | 15/09/2023 | Eligible |
| 8 | Stress | 2018 | Case-control | 423 | Mekonnen et.al | 3.31 | 2.22 | 24.09 | ND & AW | 15/09/2023 | Eligible |
|  | Stress | 2021 | Case-control | 327 | Tefera et.al | 7.01 | 2.56 | 19.18 | ND & AW | 15/09/2023 | Eligible |
|  | Stress | 2023 | Case-control | 444 | Asrade et.al | 2.19 | 1.00 | 4.79 | ND & AW | 15/09/2023 | Eligible |
|  |  |  |  |  |  |  |  |  |  |  |  |
